# Genetic Polymorphisms Associated with Fetal Hemoglobin (HbF) Levels and F-Cell Numbers: A Systematic Review of Genome-Wide Association Studies

**DOI:** 10.3390/ijms252111408

**Published:** 2024-10-23

**Authors:** Coralea Stephanou, Stephan Menzel, Sjaak Philipsen, Petros Kountouris

**Affiliations:** 1 Molecular Genetics Thalassemia Department, The Cyprus Institute of Neurology and Genetics, Nicosia 2371, Cyprus; petrosk@cing.ac.cy; 2 School of Cancer & Pharmaceutical Sciences, King’s College London, London SE5 9NU, UK; 3 Department of Cell Biology, Erasmus MC, 3015 GD Rotterdam, The Netherlands

**Keywords:** fetal hemoglobin (HbF), F cells, GWAS, SNP, genetic modifier, beta thalassemia, sickle cell disease (SCD), systematic review

## Abstract

Elevated fetal hemoglobin (HbF), which is partly controlled by genetic modifiers, ameliorates disease severity in β hemoglobinopathies. Understanding the genetic basis of this trait holds great promise for personalized therapeutic approaches. PubMed, MedRxiv, and the GWAS Catalog were searched up to May 2024 to identify eligible GWAS studies following PRISMA guidelines. Four independent reviewers screened, extracted, and synthesized data using narrative and descriptive methods. Study quality was assessed using a modified version of the Q-Genie tool. Pathway enrichment analysis was conducted on gene lists derived from the selected GWAS studies. Out of 113 initially screened studies, 62 underwent full-text review, and 16 met the inclusion criteria for quality assessment and data synthesis. A total of 939 significant SNP-trait associations (*p*-value < 1 × 10^−5^) were identified, mapping to 133 genes (23 with overlapping variant positions) and 103 intergenic sequences. Most SNP-trait associations converged around *BCL11A* (chr.2), *HBS1L-MYB*, (chr.6), olfactory receptor and beta globin (*HBB*) gene clusters (chr.11), with less frequent loci including *FHIT* (chr.3), *ALDH8A1*, *BACH2*, *RPS6KA2*, *SGK1* (chr.6), *JAZF1* (chr.7), *MMP26* (chr.11), *COCH* (chr.14), *ABCC1* (chr.16), *CTC1*, *PFAS* (chr.17), *GCDH*, *KLF1*, *NFIX*, and *ZBTB7A* (chr.19). Pathway analysis highlighted Gene Ontology (GO) terms and pathways related to olfaction, hemoglobin and haptoglobin binding, and oxygen carrier activity. This systematic review confirms established genetic modifiers of HbF level, while highlighting less frequently associated loci as promising areas for further research. Expanding research across ethnic populations is essential for advancing personalized therapies and enhancing outcomes for individuals with sickle cell disease or β-thalassemia.

## 1. Introduction

Beta (β) hemoglobinopathies are common inherited disorders of global significance, marked by substantial morbidity and mortality due to either defective hemoglobin (Hb) production, as in sickle cell disease (SCD), or insufficient production of the β-globin subunit of Hb, as seen in β-thalassemia [1]. A 2008 WHO study provided estimates of over 330,000 affected births per year (83% SCD, 17% thalassemias) [2], while recent updates from the Global Burden of Disease (GBD) 2021 indicated a global rise in SCD births by 13.7%, primarily attributed to population growth in the Caribbean and western and central sub-Saharan Africa [3]. Although comprehensive global statistics for β-thalassemia are unavailable, systematic assessment of population-based data underscores the substantial incidence of β-thalassemia across Europe, the Middle East, and Asia [4,5]. These Hb disorders constitute clinically diverse syndromes, with the severest forms leading to long-term disability and, without clinical intervention, even death. The patients also present variable responses to intervention, placing a further substantial burden on healthcare resources. Their phenotypic diversity is partly explained by genetic factors at the *HBB* (β-globin) gene locus and elsewhere in the genome, which underscores the importance of tailoring personalized therapeutic approaches. Despite decades of research, there is still a lack of comprehensive understanding of the genetic factors influencing clinical severity. While raised levels of fetal hemoglobin (HbF) are recognized as a major disease modifier, their precise genetic basis has yet to be fully elucidated. This work is indicated for the International Hemoglobinopathy Research Network (INHERENT) [6] and aims to assess all currently reported genetic modifiers of HbF levels identified at genome-wide significance in β hemoglobinopathies and the general population.

The molecular defects in the *HBB* gene (encoding β-globin) manifest clinically as HbF (α_2_γ_2_) is gradually replaced by adult hemoglobin (HbA, α_2_β_2_) around birth. The fetal-to-adult Hb switch relies on the silencing of HbF expression, which is mechanistically controlled by a complex interplay of regulatory elements, transcription factors, and epigenetic modifications, yet it is not total or irreversible [7]. In healthy individuals, HbF continues to be synthesized beyond early childhood at residual levels (<1% of total Hb) with considerable variation between individuals [8]. In SCD, HbF levels can increase up to 30% of total Hb [9], displaying variability across different *HBB* haplotypes, and with wide dispersion within each haplotype [10]. The augmentation of HbF levels, measured either as a percentage or as the number of erythrocytes with detectable HbF (termed F-cells) [11], has no clinical consequences in healthy individuals but modulates disease severity in β hemoglobinopathies. This is supported by observations of improved disease outcomes in individuals with β hemoglobinopathies who also carry genetic variants contributing to hereditary persistence of fetal hemoglobin (HPFH), a benign condition characterized by elevated HbF in adult erythroid cells [12]. HbF prevents the polymerization of sickle Hb, which is central to SCD pathogenesis and compensates for the HbA deficiency in β-thalassemia. The reactivation of HbF presents a universal approach that can be effective for the treatment of both SCD and β-thalassemia with several genomic tools and therapeutic approaches currently under investigation [13,14].

Genome-wide association studies (GWAS) constitute a powerful tool for identifying associations between common genomic variants, genotyped either using arrays or sequencing strategies, and a trait of interest [15].

Twin studies [16] and early genome-wide scans [17] revealed that the variable HbF increase in healthy adults but also individuals with β hemoglobinopathies is inherited as a quantitative genetic trait. Among the identified top hits were several genetic variants in two genomic loci not linked to the *HBB* locus: *BCL11A* on chromosome 2p16 [18] and the intergenic region between *HBS1L* and *MYB* (termed *HMIP*) on chromosome 6q23 [19]. These discoveries were quickly replicated by other studies across various populations. Together, these three loci explain up to 50% of the heritable variation in HbF [20], leaving the question open on the remaining heritability. This may include contributions of rare large effect variants, or hundreds to thousands of variants of small effects, each individually difficult to detect within GWAS [21]. 

As sample sizes increase, the number of identified GWAS-significant variants steadily expands but, even then, only the most robust variant associations can be identified with meaningful certainty. It is often the case that a GWAS-significant variant does not cause the observed phenotypic effect directly but is in linkage disequilibrium with the one that does, residing within the same genomic locus. GWAS signals may also be associated with other traits, posing a challenge for interpreting the GWAS outcome in a biological context [15].

Despite the increasing number of quantitative trait GWASs conducted for elevated HbF in the past decade, a systematic summary of these findings has been lacking. As the number of novel, often mechanistically unexplained, genomic loci continues to rise across individual GWASs that may differ in statistical methods and with heterogeneous or overlapping sampling, the insight added by each new GWAS is often not immediately evident. A comprehensive analysis of the literature by INHERENT highlights the absence of ancestral diversity in GWAS and the constrained range of disease genotypes in β hemoglobinopathies, with most GWASs focused on SCD within African-Americans [6]. This limits the discovery of HbF-modulating variants to those present in this specific population and under-utilizes the potential to make new genetic discoveries inherent to every additional human ethnic group under study. Additionally, differences in allele frequencies for single-nucleotide polymorphisms (SNPs) and gene-trait associations across ancestries further complicate direct comparisons between groups of individuals. Consensus is yet to be reached on which genomic variations significantly contribute to HbF heritability and which ones are relevant to specific populations. Therefore, caution should be exercised in interpreting the findings of these GWASs. An effective starting point in post-GWAS analyses involves looking for convergence of GWAS-significant variants on specific genes by conducting pathway or gene-set enrichment analysis. A critical appraisal of the papers supporting gene-trait associations, particularly examining evidence of replication, is necessary, as such findings can be used to devise population-oriented genetic scoring systems to predict the disease trait in question [22,23] and guide personalized therapeutic applications [24]. In line with existing literature, this is the first systematic review to comprehensively address genetic modifiers of HbF levels on a global scale, providing an up-to-date analysis of all available evidence. It extends beyond the well-characterized targets of *BCL11A*, *HBS1L,* and the *HBB* locus, emphasizing the need for further research across diverse ancestries and disease groups. This systematic review is the first to summarize the current literature, assess the quality of the findings, and report on the areas that need to be addressed towards the identification of promising candidates for HbF reactivation as a therapeutic approach for patients with β hemoglobinopathies.

## 2. Materials and Methods

### 2.1. Registration of Protocol and Reporting

The systematic review protocol was registered on the International Prospective Register of Systematic Reviews (PROSPERO CRD42023415900), and the review process was conducted in line with the Preferred Reporting Items for Systematic Reviews and Meta-Analyses (PRISMA) 2020 checklist.

### 2.2. Search Strategy

The PICO framework was used to form the research question (Table 1) and guide the literature search. The search query was conducted in the electronic database PubMed using the keywords *(“genome-wide association” OR GWAS OR “genome wide association” OR “whole genome” OR “whole-genome”) AND (fetal OR “F cell” OR “F-cell” OR “HbF” OR “Hb F”) AND (hemoglobin OR sickle OR thalassemia OR globin)*, from inception up to 31 March 2023 (inclusive). A combination of keywords related to GWAS and HbF was also searched in the preprint servers bioRxiv and medRxiv to identify relevant publications submitted between 1 January 2021 and 31 March 2023. Curated collections of published GWAS were accessed from the NHGRI-EBI GWAS Catalog (data accessed on 31 March 2023). Letters and editorials were considered for insights from small-scale studies, while reviews were included for their reference lists and were hand-searched for additional relevant publications. A final literature search was conducted on 31 May 2024 to identify potentially new content prior to finalizing the manuscript.

### 2.3. Inclusion and Exclusion Criteria

Articles were included in the systematic review if they reported (i) new GWAS data, (ii) clearly defined phenotypes (HbF or F-cell content) determined using standard laboratory methods, (iii) accurate sample descriptions (size and ancestry), (iv) statistical significance (SNP-trait *p*-value < 1 × 10^−5^), (v) allele information (effect and other allele), and (vi) association size and direction (β [standard error (SE)], OR [95% confidence interval (CI)]).

Conversely, articles were excluded from the systematic review if they: (i) were published in a language other than English, (ii) reported only *p*-values without effect estimates, (iii) included SNP panels limited to candidate genes, (iv) reported β^S^ globin haplotype-trait associations, and (v) examined cohorts undergoing transfusion therapy or pharmacological treatment with HbF-inducing agents. Evidence from expert opinion commentaries, websites, and master’s or doctoral theses were also excluded from consideration.

### 2.4. Study Selection

All literature references were imported into a single electronic library in Zotero [25] and uploaded on Rayyan [26] as a single review for screening. Duplicate articles from searches were removed automatically, and the titles and abstracts of the remaining articles were screened by four reviewers (C.S., S.P., S.M., P.K.) independently using the predefined set of eligibility criteria. The full manuscript text of these potentially eligible articles, including the results and available Supplemental Materials, were retrieved and assessed by the same investigators for inclusion in the review. Any disagreements were resolved by group consensus.

### 2.5. Data Extraction

A standardized table in Microsoft Excel developed by INHERENT [6] was utilized for data extraction from the included articles by one author (C.S.), with subsequent review by S.P., S.M., and P.K. In cases where data were absent from the full manuscript text, requests were made to study authors via email for the missing information. The extracted data encompassed elements from the GWAS Catalog’s GWAS summary statistics format (GWAS-SSF) [27], as well as items specified in the PRISMA 2020 and Q-Genie checklists, including publication details, phenotype definitions, cohort descriptions (size, ancestry, β genotype), genetic variables (genetic model, allele, allele frequency), association statistics (*p*-value, β [SE], OR [95% CI]), as well as further elements crucial to GWAS experiments (genotyping methods, sample and marker quality control analysis [28], regression analysis, confounding variables). The full item list is found in Supplementary Materials Tables S1–S3.

### 2.6. Data Quality Assessment

A modified version of the Quality of Genetic Studies (Q-Genie) tool (https://fhs.mcmaster.ca/pgp/documents/Q-Geniev.1.1.pdf, accessed on 12 October 2023) was used to appraise the included studies that fulfilled the eligibility criteria. Instead of applying numerical scores to each question based on a predefined rating scale and subsequently calculating a cumulative score for each study, the reviewers employed an ordinal scale with three categories: “high risk of bias”, “some concerns”, and “low risk of bias”. The reviewers categorized the risk of bias into one of three levels based on the amount of evidence collected for each question (see Supplementary Materials Table S4 for detailed item descriptions for each appraised study). Four independent reviewers (C.S., S.P., S.M., P.K.) assessed the quality of each study, and any disparity between decisions was resolved by consensus. Decisions regarding each domain of bias were made based solely on the gathered evidence, as no algorithm was used for ranking the studies; the reviewers believe all studies have merit. These assessments were made across 11 domains of bias as defined by Q-Genie (Supplementary Materials Table S4): (a) GWAS design rationale (publication bias), (b) phenotype selection and definition (information and measurement bias), (c) sample selection including comparison groups (sampling bias), (d) sample size/power considerations, (e) technical classification of the genetic variant, including genotyping methods and quality control steps (genotype misclassification), (f) non-technical classification of the genetic variant (detection bias), (g) disclosure and discussion of other sources of bias, (h) a priori planning of analysis (selective reporting bias), (i) statistical methods and control for confounding (bias from multiple correction testing, population stratification), including replication studies and meta-analyses, (j) testing of assumptions and inferences for genetic analyses (bias from consanguinity/relatedness, sex and ethnicity discrepancies, inappropriate selection of haplotype structure), and (k) appropriateness of inferences drawn from results (reporting bias).

### 2.7. Gene Annotation

All SNPs that exceeded the statistical significance threshold of 1 × 10^−5^ (as defined in accordance with the 2010 GWAS Catalog recommendations) were extracted and included in the results table. Gene annotations for all rs_IDs in the extracted list were standardized to HGNC names using dbSNP. Intergenic SNPs were annotated to the closest upstream and downstream protein-coding genes using the NCBI Sequence Viewer 3.49.0. From this results table, a compilation was made of unique genes and intergenic regions annotated with significant results, also detailing the count (*n*) and identifiers (rs_ID) of unique SNPs associated with each gene and intergenic region.

### 2.8. Experimental Evidence-Based Gene Compilation

A list of genes standardized to HGNC names was compiled based on published experimental evidence as Hb switching factors and modulators of HbF expression. The evidence categories, as defined in [29], include experimental protein interactions, expression, functional alteration, and model systems. This list, termed “genes_E”, includes gene-level experimental evidence from publications up to December 2023. The modifier gene list in the ITHANET Portal (https://www.ithanet.eu/) [30] was accessed as an additional source of data (accessed on 29 December 2023).

### 2.9. Gene Set Enrichment Analysis and Pathway Analysis

The complete list of SNPs (Supplementary Materials Table S5) was entered into the snpXplorer web server [31] (accessed on 11 June 2024) to perform variant-to-gene mapping and functional annotation of SNPs. The server’s algorithm first links SNPs to likely affected genes and then performs gene-set enrichment analysis to identify annotations that are overrepresented among the input SNPs. This process aids in determining which genes may be influenced by the genetic variants and their significance within biological contexts. Additionally, variant-gene mapping and SNP annotation were conducted on a subset of SNPs located in genes and intergenic regions identified in two or more independent publications.

Next, the gene set generated from snpXplorer for this subset of selected SNPs was used as input for g:Profiler [32] (accessed on 11 June 2024) to interpret the biological significance and identify molecular pathways enriched with genes harboring the input SNPs. Pathway analysis was also performed on a set of genes supported by experimental evidence (as described in Section 2.8).

### 2.10. Gene Constraint Metrics

Gene constraint scores for the predicted loss of function (pLoF) metric were obtained from gnomAD v4.1.0 [33]. The degree of pLoF constraint was measured by two metrics; the probability of being LoF intolerant (pLI, with a cutoff ≥ 0.9) and the observed/expected ratio (o/e) of the LoF effect predictor (LOEUF, with a cutoff < 0.35) [34]. A higher pLI score (i.e., observing fewer LoF variants than expected) indicates higher constraint, meaning that the gene is more likely to be intolerant to loss-of-function variants (i.e., depletion of variation). Gene constraint scores help assess how sensitive a gene is to genetic changes, with higher scores indicating that the gene is less tolerant to such changes and more likely to be associated with health problems. A two-tailed *t*-test was performed using the T-TEST function in Microsoft Excel to compare the means of the pLI scores and o/e scores between the two groups (‘genes_G’ vs. ‘genes_E’ as detailed in Supplementary Materials Table S6). Prior to the *t*-test, an F-test was calculated using the F.TEST function in Microsoft Excel, which indicated that the variances were significantly different, supporting the use of a heteroscedastic *t*-test. A *p*-value < 0.05 was used to indicate the statistical significance of the observed differences.

## 3. Results

### 3.1. Study Selection

**Figure 1:**
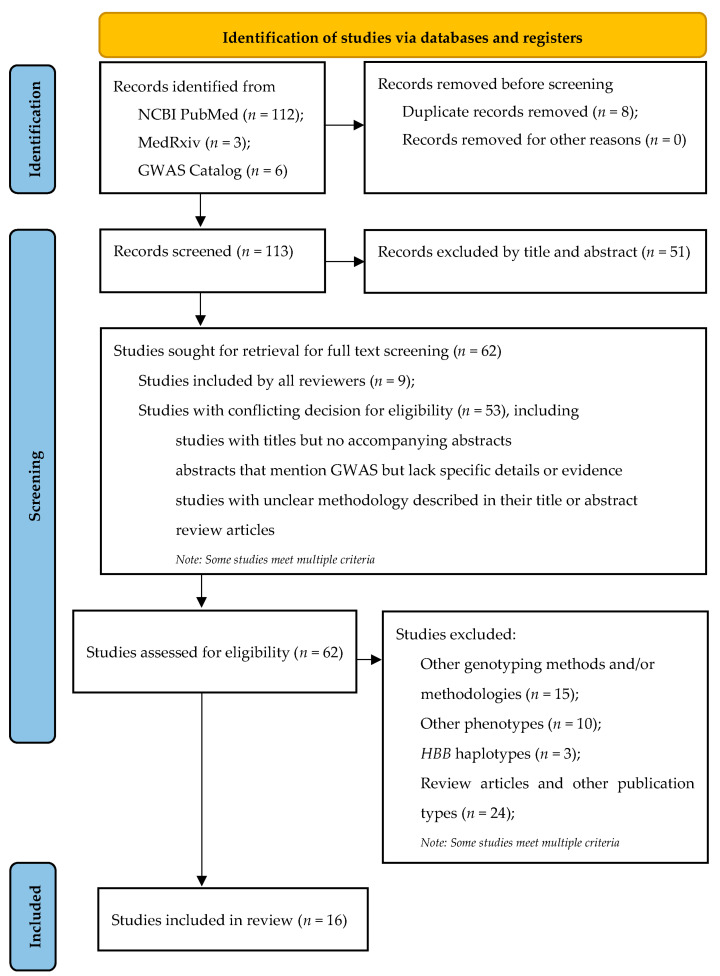
PRISMA flow diagram.

During the initial literature search, a total of 121 records was identified; this number was reduced to 113 unique records after removing duplicates. Subsequently, after screening titles, abstracts, and full texts, studies that did not meet the eligibility criteria were excluded. In cases where studies did not provide published data in the results and Supplementary Materials, the study authors were contacted for clarification. This thorough process resulted in a final selection of 16 studies for inclusion in the analysis. Of note, all six studies [17,35,36,37,38,39] obtained from the GWAS Catalog were also identified in the PubMed search, thus lending support to the appropriateness of the search strategy. A flow diagram of the selection and screening process of these studies is shown in Figure 1.

### 3.2. Study Characteristics

The final list of 16 papers included 14 distinct GWASs [17,18,35,37,38,39,40,41,42,43,44,45,46,47] and two meta-analyses that combined cohort-specific GWAS data generated anew [48,49]. Except for three small studies that evaluated genetic associations with F-cell numbers [17,18,38], the remaining studies, including the two meta-analyses, examined the molecular basis of HbF heterogeneity. Most of these studies had sample sizes of fewer than 2000 individuals, although there were notable exceptions, including three studies with European (non-hemoglobinopathy) cohorts (*n* = 4305 [37], 6602 [43] and 11,004 [46]), and a study with 2040 [49] SCD individuals with African ancestry. Cohorts from trials that reported results on ClinicalTrials.gov were utilized in more than one publication, usually in conjunction with other same-ancestry cohorts to increase sample size, a notable example being the Cooperative Study of Sickle Cell Disease (CSSCD; clinicaltrials.gov #NCT00005277). The largest sample size of nearly 30,000 individuals, involving both SCD and non-hemoglobinopathy samples obtained from several distinct cohorts of diverse ancestries (European, African, Thai), was reported in the meta-analysis by Cato et al. [48]. Moreover, studies with unselected individuals from the general population (non-hemoglobinopathy) mainly involved cohorts of European ancestry, whereas studies specifically addressing individuals with β hemoglobinopathies focused on sickle cell disorders of any genotype (SS, Sβ0) and were conducted among samples of African ancestry, predominantly in African American cohorts. Only two studies delved into genetic modifiers of SCD within African individuals, one conducted in East Africa (Tanzania, *n* = 1213) [42] and another in West Africa (Nigeria, *n* = 1006) [47]. Furthermore, three studies involved Asian cohorts (Thai, Chinese/Hong-Kong) with β-thalassemia trait or HbE trait [18,39,40], albeit in small numbers, yet no study was conducted in β-thalassemia. All studies were inclusive of gender and age groups (child, adult), and provided pre-transfusion HbF or F-cell measurements at the age of 5 years or older, except for two studies [44,45], which adhered to an age cutoff of 2 years old. The characteristics and details of the included studies are summarized in Table 2, with additional data in Supplementary Materials Tables S1 and S2.

### 3.3. Critical Appraisal of Included Studies

The quality of each study was evaluated using a modified version of the Q-Genie tool, categorizing studies as “high risk of bias”, “some concerns”, and “low risk of bias”. Table 3 provides a descriptive assessment of study quality and potential biases, highlighting the strengths and weaknesses of each included study (refer to Supplementary Materials Table S4 for a detailed appraisal of the papers). All 16 studies described GWAS-significant genetic variants associated with HbF or F-cell numbers based on a priori hypotheses, guided by the design of the genotyping arrays and analysis methods. The majority of studies (12/16, 75%) utilized reliable laboratory methods to measure the phenotype, and while most of these studies adequately described the sample population, only a subset (4/16, 25%) performed power analysis to substantiate their chosen sample size. Additionally, a few studies, primarily among the initial GWASs in this field, were designated as having a “high risk of bias” concerning questions about the quality control of GWAS data and statistical analyses due to the absence of methodological details. More recent studies, benefiting from technological advances and data imputation methods, were categorized as having a “low risk of bias” across most domains of bias. All 16 studies were included for analysis since they have reported GWAS-significant associations successfully.

### 3.4. Genetic Associations Per Disease Status

All 16 included studies reported outcomes that reached statistical significance (SNP-trait *p*-value < 1 × 10^−5^), with a total of 939 GWAS-significant SNP-trait associations (Supplementary Materials Tables S2 and S3). Among these, 552 were reported in SCD, 19 in β-thalassemia trait, 77 in the general population (non-hemoglobinopathy), and 291 in mixed cohorts in two large meta-analyses. Most SNP-trait associations in disease cohorts were mapped to *BCL11A* and the *HMIP* region, contrasting with findings from the general population, which revealed additional molecular signatures. This suggests that *BCL11A* and *HMIP* may have a greater impact on modulating the HbF phenotype in disease cohorts, while general population studies provide insights into the role of genetics in overall health. The meta-analyses, which involved larger sample sizes, identified a broader range of candidate genetic modifiers associated with HbF levels, underscoring the complexity of the genetic landscape influencing HbF, particularly when compared to individual studies in SCD populations.

#### 3.4.1. GWAS Findings in SCD Cohorts

Ten studies investigated genetic variants associated with changes in the percentage of HbF (%[HbF], *n* = 9) or F-cells (%[F-cells], *n* = 1) within SCD cohorts (SS or SS and Sβ0 genotypes), reporting a total of 552 SNP-trait associations (430 unique dbSNP IDs and eight sequence variations without an rs identifier). The majority of SNPs (*n* = 244) resided within intergenic sequences (79 unique regions), while 192 SNPs were mapped across 80 protein-coding genes. The most significant associations included rs1427407 (allele: G; *p*-value: 3.79 × 10^−53^; β: −0.30) [42] and rs6706648 (allele: C; *p*-value: 4.96 × 10^−34^; β: −0.395) [47] in *BCL11A* and rs7606173 in the *HMIP* region (allele: G; *p*-value: 8.50 × 10^−33^; β: −0.393) [47]. The full list is available in Supplementary Materials Table S2.

Furthermore, 159 SNP-trait associations were replicated across studies, involving 62 unique associations with either %[HbF] or %[F-cells] across 45 SNPs. The majority of the replicated SNP-trait associations (*n* = 24) were identified in two studies. Upon selecting the study with the most statistically significant *p*-value from independent GWASs with overlapping cohorts, the count of replicated SNPs was reduced to 31. All had an association with a change in %[HbF], with 17 also showing an association with a change in %[F-cells]. These replicated SNPs converged around two genomic loci: *BCL11A* (23 SNPs) and the *HMIP* region (7 SNPs). One SNP mapped to the unsuspected locus *FHIT* (chr.3). *BCL11A* demonstrated the most consistently replicated findings, specifically for rs766432 (eight cohorts) and rs10195871 (seven cohorts). Furthermore, the most significant *p*-values were reported for SNPs associated with %[HbF] in *BCL11A*, including rs1427407 (allele: G; *p*-value: 3.79 × 10^−53^; β: −0.3) [42], rs6706648 (allele: T; *p*-value: 4.96 × 10^−34^; β: −0.395) [47], and rs7606173 (allele: C; *p*-value: 8.50 × 10^−33^; β: −0.393) [47]. Notably, rs7606173-*BCL11A* also had the most significant association with %[F-cells] (allele: G; *p*-value: 5.14 × 10^−16^; β: 1.5) [38].

These findings underscore the importance of *BCL11A* influencing both %[HbF] and %[F-cells] in the studied SCD cohorts.

#### 3.4.2. GWAS Findings in Cohorts with Beta Thalassemia Trait

Two GWASs identified a total of 19 SNP-trait associations with %[HbF] in individuals with heterozygous β-thalassemia (*n* = 18) or hemoglobin E (HbE; *n* = 1) [39,40]. Both studies utilized overlapping samples of Hong Kong Chinese with heterozygous β-thalassemia. rs766432 in *BCL11A* replicated across both studies, reducing the number to 18 unique SNPs. These SNPs were located on chromosomes 2 and 6, with six SNPs mapping in *BCL11A*, one SNP in the *MIR4432HG-BCL11A* region (chr.2), 10 SNPs in the *HMIP* region, and one SNP in *ALDH8A1* (chr.6). The most significant associations were found in the *HMIP* region with rs9399137 (allele: T; *p*-value: 1.39 × 10^−24^; β: 0.45) and rs7775698 (allele: C; *p*-value: 1.38 × 10^−23^; β: 0.44).

#### 3.4.3. GWAS Findings in the General Population

A total of 77 SNP-trait associations were reported in four European population-based studies. Three studies showed associations with changes in HbF (% or g/dL) and one study with changes in %[F-cells]. Among these, three SNPs were associated with both traits across two studies: rs1427407 and rs4671393 in *BCL11A* and rs9399137 in the *HMIP* region. Most of the 74 GWAS-significant SNPs are located on chromosomes 2 (32%, 24/74), 11 (38%, 28/74), and 6 (16%, 12/74).

SNPs on chromosome 2 clustered in *BCL11A* (*n* = 10), *MIR4432HG* (*n* = 9), and the genomic sequence between them (*n* = 4), while SNPs on chromosome 6 clustered in the *HMIP* region (*n* = 8), with one SNP each on *HBS1L*, *ALDH8A1* and *BACH2*. SNPs on chromosome 11 mainly spanned the *HBB* locus and flanking olfactory genes, and three SNPs mapped on *UBQLNL*. Other loci that emerged in the analysis with one or two significant SNPs included *ARHGAP39* (chr.8), *ABCC1* (chr.16), *PFAS* and *CTC1* (chr.17), *GCDH* and *NFIX* (chr.19), and *CSNK2A1* (chr.20). The strongest association was reported for rs7776054 in *BCL11A* with %[HbF] (allele: G; *p*-value: 9.40 × 10^−164^; β: 0.51) [46].

#### 3.4.4. Meta-Analysis Findings

A large HbF meta-analysis, encompassing 29,279 individuals of mixed ancestry (European, African, Asian/Thai) and various disease states (SCD and non-hemoglobinopathy) identified 77 SNP-trait associations at genome-wide significance [48]. This study represents the most extensive meta-analysis conducted thus far to explore genetic modifiers of %[HbF], with major genomic loci such as *BCL11A*, the *HMIP* region, *HBB*, and *CTC1* identified across the entire cohort.

Moreover, a multi-ancestry meta-analysis unveiled 69 SNP-trait associations specifically among individuals with SCD (comprising 3963 samples from African, African-American, and Brazilian ethnic backgrounds) [48]. In a separate meta-analysis focused on SCD, involving 2040 African-American samples, 23 SNP-trait associations were reported at a *p*-value < 1 × 10^−5^ [49]. Two SNPs in *BCL11A* (rs6706648 and rs6709302) and one SNP in the *HMIP* region (rs9494145) were replicated in both studies, resulting in a total of 89 unique SNP-trait associations in African ancestry populations with SCD. A large proportion of these SNPs (49.4%, 44/89) were mapped in chromosome 11, spanning the *HBB* cluster (19 SNPs), the olfactory receptor gene cluster flanking the *HBB* cluster (22 SNPs), and with one SNP each in *CARS1*, *MMP26*, and *TRIM5*. Additionally, a substantial number of SNPs were located on chromosome 2 (21.3%, 19/89) within *BCL11A* (13 SNPs), *MIR4432HG* (one SNP), and the region between them (four SNPs), as well as on chromosome 6 (19.1%, 17/89), mainly within the *HMIP* region (12 SNPs), the *SGK1-ALDH8A1* region (three SNPs), and one SNP each in *MYB* and *BACH2*. Other genomic loci included *PFAS* and its surrounding region (chromosome 17, five SNPs) and *SGCZ* (chromosome 8, one SNP).

### 3.5. Exploring Genes within the Dataset

The 939 GWAS-significant SNP-trait associations (*p*-value < 1 × 10^−5^) across the 16 included studies mapped to 133 genes, of which 23 genes were identified as overlapping genes harboring significant SNPs, along with 103 intergenic sequences. Table 4 provides a summary of genes along with the unique rs_IDs for GWAS-significant SNPs, identified by at least two independent studies; for the complete list, we refer to Supplementary Materials Table S7. Notable among these findings was *BCL11A*, with 60 SNPs identified across all 16 studies, along with an additional 16 SNPs found upstream (*MIR4432HG-BCL11A*, three studies) and six SNPs downstream (*BCL11A-PAPOLG*, one study) of *BCL11A*. The *HBS1L-MYB* region (*HMIP*) emerged in 12 studies with 41 SNPs. Individual genes *HBS1L* (with 3 SNPs) and *MYB* (with six SNPs) were identified in two and one study, respectively.

*OR51B5* was identified with 32 SNPs, some of which overlapped with other genes, across six studies. An additional 118 SNPs were found across four studies in the olfactory receptor gene cluster, with the majority mapping to *OR52A1-OR51V1* (35 SNPs), *OR51B4-OR51B2* (16 SNPs), and *OR51B2-OR51B5* (12 SNPs) loci. The *HBB* cluster appeared with 32 SNPs in eight studies, along with an additional 16 intergenic SNPs with olfactory receptor genes in four studies.

*MIR4432HG*, along with upstream (*FANCL-MIR4432HG*, five SNPs) and downstream (*MIR4432HG-BCL11A*, 16 SNPs) regions, emerged in five studies with a total of 38 SNPs.

Notable genomic loci that emerged in three studies featured *MMP26* (chr.11) with 19 SNPs, some of which overlapped with olfactory receptor genes, *ALDH8A1* (chr.6) with six SNPs, and *FHIT* (chr.3) with two SNPs. *ALDH8A1* also appeared with intergenic SNPs with *HBS1L* (one SNP; chr.6) and *SGK1* (nine SNPs; chr.6) in one study. Other loci appearing in two studies included *CSNKA2IP-EPHA3* (22 SNPs; chr.3), *CTC1* (six SNPs; chr.17), *PFAS* (six SNPs; chr.17), *ABCC1* (four SNPs; chr.16), *BACH2* (four SNPs; chr.6), *GCDH* (three SNPs; chr.19), *COCH* (one SNP; chr.14), *NFIX* (one SNP; chr.19) and *RPS6KA2* (one SNP; chr.6). Other noteworthy loci appearing in one study, albeit with functional data in literature, included *JAZF1* (one SNP; chr.7) [69], *KLF1* (one SNP; chr.19) [70] and *ZBTB7A* (one SNP; chr.19) [71], as well as *PTEN* (one SNP; chr.10) [72] and *SGK1* (nine SNPs; chr.6) [73] as part of intergenic SNPs.

### 3.6. Gene Enrichment and Pathway Analysis

#### 3.6.1. Gene Mapping and Functional Annotation of SNPs

**Figure 2:**
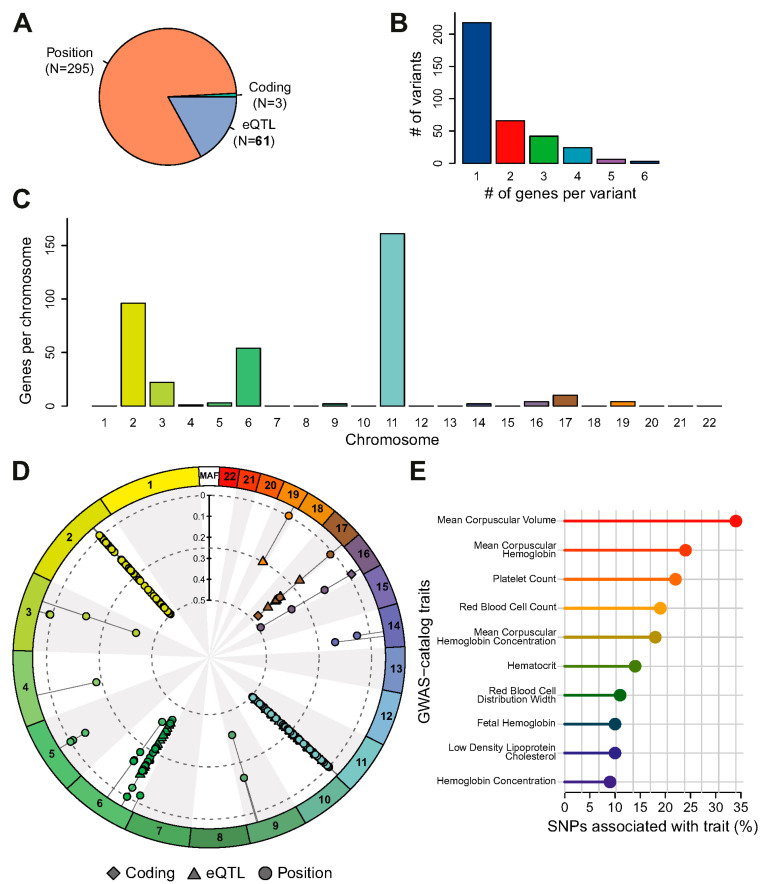
Gene mapping and functional annotation of the selected SNP set (found in ≥two papers) using snpXplorer. (**A**) Pie plot shows variant annotations, classified as coding (green), eQTL (blue), or annotated by their genomic position (orange). It informs about variant effect, i.e., a direct effect on protein sequence in the case of coding SNPs, or a regulatory effect in the case of eQTLs or intergenic SNPs. (**B**) Barplot shows the number of genes associated with each SNP. Variant-gene mapping might associate multiple genes to a single SNP, depending on the effect and position of each SNP. (**C**) Plot shows the chromosomal distribution of SNPs. (**D**) The circular summary figure shows the type of annotation of each SNP used as input (coding, eQTL, or annotated by their positions) as well as each SNP’s minor allele frequency (MAF) and chromosomal distribution (numbers 1–22 on the outer ring). (**E**) Fraction of SNPs associated with traits from the GWAS Catalog, estimated by averaging fractions across 500 iterations of sampling one gene per variant to correct for multiple genes being associated with a single variant.

The variant-to-gene mapping analysis in snpXplorer, using the complete list of SNPs (*n* = 707, Supplementary Materials Table S5), revealed a wide range of likely affected genes across the genome, with notable clusters on chromosomes 2, 6, 11, and 19 (Supplementary Materials Figure S1). When using the selected SNPs (found in ≥two studies) as input data (*n* = 366, Supplementary Materials Table S5), the number of likely affected genes was markedly reduced, primarily located on chromosomes 2, 6, and 11. Additionally, likely affected genes were observed on chromosomes 3, 4, 5, 9, 14, 16, 17, and 19, albeit with a lower number of SNPs (Figure 2C,D). The server’s algorithm may associate multiple genes with a single SNP depending on the effect and position of each SNP, circumventing the challenge in functionally interpretating SNPs located in non-coding regions of the genome where establishing a direct mapping between genetic variants and affected genes is often complex [31]. Functional annotation using as input the list of selected SNPs resulted in a set of traits (provided that the SNPs and/or genes were previously associated with any trait in the GWAS Catalog) that included mostly hematological traits such as ‘mean corpuscular volume’, ‘mean corpuscular hemoglobin’, ‘platelet count’, and ‘fetal hemoglobin measurement’ (Figure 2E). Downstream pathway analysis was performed using the list of selected SNPs and their linked genes from the snpXplorer analysis.

#### 3.6.2. Functional Annotation of Genes

**Figure 3:**
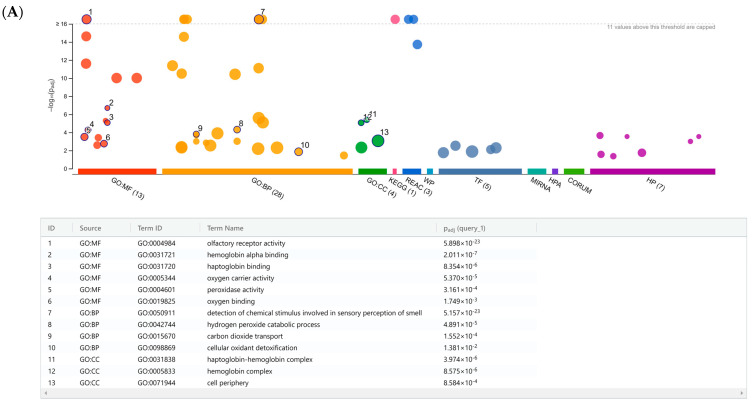

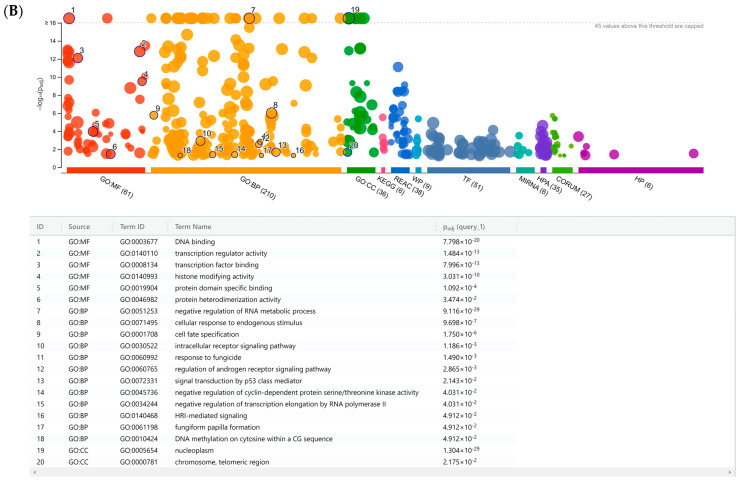
Enrichment analysis in g.Profiler. (**A**) Gene set from selected SNP list (‘genes_G’); (**B**) Gene set compiled from experimental evidence (‘genes_E’).

Given the prominence of the olfactory receptor gene cluster in the selected SNP input (‘genes_G’, Supplementary Materials Table S6), it comes as no surprise that genes provided to g:Profiler were enriched for GO terms and pathways involved in olfaction, including GO terms olfactory receptor activity [MF GO:0004984] and detection of chemical stimulus involved in sensory perception of smell [BP GO:0050911], and pathways olfactory transduction [KEGG:04740], expression and translocation of olfactory receptors [REAC:R-HSA-9752946], olfactory signaling pathway [REAC:R-HSA-381753], and sensory perception [REAC:R-HSA-9709957], Figure 3A (full list in Supplementary Materials Table S8). The GO terms enriched for genes supported by experimental evidence (‘genes_E’, Supplementary Materials Table S6) included DNA binding [MF GO:0003677], negative regulation of RNA metabolic process [BP GO:0051253] and nucleoplasm [CC GO:0005654], and pathways Polycomb repressive complex [KEGG:03083] and Gene expression (Transcription) [REAC:R-HSA-74160], Figure 3B (full list in Supplementary Materials Table S9). Enrichment analysis of the combined dataset from both gene lists revealed GO terms and pathways identified in both analyses, with the most prominent terms associated with olfaction and transcription activity (full list in Supplementary Materials Table S10).

#### 3.6.3. Gene Constraint Metrics for Genes

Most of the genes linked to the selected SNPs (‘genes_G’ list, *n* 57) had pLI scores close to zero (pLI: 0.2 ± 0.34 [mean ± SD], 0.01 [0–1] [median (min–max)]; o/e: 0.69 ± 0.55 [mean ± SD], 0.65 [0–2.96] [median (min–max)]), whereas genes supported by experimental evidence (‘genes_E’ list, *n* 58) had higher pLI scores close to 1 (pLI: 0.68 ± 0.45 [mean ± SD], 1 [0–1] [median (min–max)]; o/e: 0.3 ± 0.25 [mean ± SD], 0.23 [0–1.35] [median (min–max)]), Supplementary Materials Table S6. The *t*-test results indicate that the observed differences between the means of the pLI scores (*p*-value: 3.03358 × 10^−9^) and the o/e scores (*p*-value: 5.41203 × 10^−6^) in the two groups (‘genes_G’ vs. ‘genes_E’) are statistically significant, suggesting that these differences in constraint scores are highly unlikely to be due to random chance. Genes linked to SNPs identified through GWAS experiments have an overall low constraint score (pLI < 0.9 and LOEUF > 0.35), indicating that these genes have more variants observed than expected and are thus tolerant to variation. This is to be expected, as GWAS identifies common variations that most likely tag regions of linkage disequilibrium containing the causal variations. Most genes in the experimental list were not identified by GWAS, likely due to their intolerance to genetic variation; however, five genes appeared in both lists: *HBG1*, *HBG2*, *BCL11A*, *MYB*, and *NFIX*. These lists are not exhaustive, and other GWAS genes supported by experimental evidence may be known.

## 4. Discussion

HbF is widely acknowledged as a key ameliorating factor of disease severity in β hemoglobinopathies and a promising target for personalized therapeutics in both β-thalassemia and SCD. Despite the identification of a few genetic loci as major modulators of HbF levels, there remains a considerable gap in understanding the remaining heritability components driving this phenotypic expression of HbF. This systematic review identified 16 GWASs conducted between 2007 to 2023, spanning varying quality levels from low to high bias according to the Q-Genie tool, and reporting GWAS summary statistics on HbF levels or F-cell numbers from diverse ethnic groups. The lower risk for bias for studies published in more recent years reflects improvements in research practices over time and highlights advancements in publication standards within the field of genetic studies.

The studies included in this systematic review collectively describe 939 SNP-trait associations at a *p*-value < 1 × 10^−5^. Notably, only five studies provided a comprehensive overview of genetic associations by including both significant and non-significant findings from GWAS analyses in their results [38,39,45,48,49] (Supplementary Materials Table S2). Variations in methodologies, including data transformation approaches and reporting standards, particularly those excluding non-significant GWAS results, posed a challenge in synthesizing results across studies for a meta-analysis. Despite this limitation, stemming from publication bias across most studies, the rigorous systematic review of GWAS data validated known genetic modifiers and expanded our knowledge of the genetic contributions to changes in HbF levels and/or F-cell numbers.

As anticipated, the *HBB* cluster, *BCL11A*, and the *HBS1L-MYB* intergenic region were consistently found to be associated with HbF changes in the included studies across all disease groups (i.e., non-hemoglobinopathy, SCD, β-thalassemia trait), involving a substantial number of SNPs. Remarkably, the XmnI-*HBG2* variant (rs7482144), documented in literature as a significant modulator of HbF (see ITHANET variant record ithaID = 2127), was observed in only one GWAS study, correlating with the F-cell phenotype [17]. This observation reinforces previous reports suggesting that the strong association signal on 11p15 within the *HBB* cluster is likely attributed to functional elements in linkage disequilibrium with rs7482144 [74]. Moreover, the most significant associations were reported in meta-analyses and GWASs conducted in the general population, likely attributed to their utilization of large sample sizes, often in the thousands. These studies further expand the coverage of candidate variants for gene mapping, potentially accounting for a portion of the missing heritability of HbF. Notable examples of genes include *KLF1*, *NFIX*, *ZBTB7A*, and *BACH2* reported only in one or two of the studies included.

At a closer look, *KLF1*, identified through linkage analysis of a Maltese HPFH family, encodes a transcription factor essential for the expression of virtually all erythroid genes. In adult erythroid cells, *KLF1* preferentially activates *HBB* over *HBG1* and *HBG2* expression and directly activates the HbF repressor *BCL11A*. HPFH individuals in the Maltese family carried a stop codon variant (p.K288X), annotated as SNP rs267607202 (absent from gnomAD full exome and genome databases), on one KLF1 allele, reducing functional KLF1 levels. This variant affects BCL11A expression, leading to less efficient repression of the γ-globin genes and suggesting a molecular mechanism for the HPFH phenotype [75]. This variant has not been captured in GWASs. In fact, as GWASs measure the average effects of alleles across thousands of individuals, they are most likely to miss the unique effect sizes detected in family-level studies, which are better at identifying rare and null variants [76]. Additionally, since MYB was found to activate the *KLF1* gene, a genetic regulatory network (GRN) for hemoglobin switching comprising MYB, KLF1, and BCL11A converging on the *HBB* gene cluster, was proposed. This core GRN has now been expanded with additional transcription factors including ZBTB7A and NFIX, and coregulators including DNMT1, EHMT1/2, and components of the nucleosome remodeling and deacetylase (NuRD) complex [77]. A broad arsenal of molecular biology tools has been applied to discover novel HbF regulators. Notably, the use of immortalized human erythroid progenitor cells first reported in 2013 as a model for adult erythropoiesis [78], has been instrumental for the success of CRISPR/Cas-mediated genetic screens aimed at elucidation of the in cis and in trans HbF regulatory network. Currently, over 45 trans-acting factors have been implicated in hemoglobin switching, mainly via functional studies. It is important to realize that the majority of these have as yet not been associated with HPFH in humans and might never be. Detection of important genes through human genetic studies requires that such genes harbor functionally significant DNA polymorphisms and also that the effect of such variants is compatible with embryonal survival. Our analysis further supports this by demonstrating statistical differences in constraint scores between genes identified by GWAS and genes supported by experimental evidence. It is therefore not surprising that the gene list compiled based on experimental evidence mainly includes constrained genes, which are tagged by GO terms and pathways associated with transcriptional regulation, a critical biological process that defines cell identity and function. Thus, additional dedicated GWAS studies, such as those proposed by INHERENT, and focused studies on HPFH families and sporadic cases are needed to bridge this gap.

Furthermore, the functional annotation and pathway enrichment analysis of GWAS genes highlighted terms related to olfaction, mainly due to the prominent presence of olfactory receptor region SNPs within the dataset. The *HBB* cluster is surrounded by genes encoding olfactory receptors, which are typically organized into inactive (DNase resistant) chromatin. This chromatin changes conformation during erythroid cell differentiation in a process controlled by the LCR. It was initially proposed that the olfactory receptor gene region might control the expression of the *HBB* cluster, as it contains a DNase I hypersensitive site and binding sites for the erythroid-specific transcription factors GATA1 and KLF1 [79]. Additionally, CTCF binding sites (CBSs), which anchor chromosomal loops in the *HBB* cluster to regulate globin gene expression, are located at the borders of the β-globin gene cluster (3′HS1 and 5′HS5) and in the surrounding region of olfactory receptor genes (3′ OR52A5 and 5′ OR51B5). Disruption of CBSs alters chromatin organization in the *HBB* cluster and has revealed an HPFH enhancer between the 3′HS1 and 3′-OR52A5 CBSs, located in the *OR52A1* gene. This enhancer contributes to the regulation of *HBG2* and *HBG1* gene expression [80], and its flanking olfactory receptor genes are identified in the GWAS. These findings underscore the potential importance of the olfactory receptor gene region as a distinct regulatory locus, independent of the *HBB* cluster.

Additionally, significant molecular function terms identified in this analysis further include ‘hemoglobin alpha binding’ and ‘haptoglobin binding’, which are associated with mechanisms of oxidative stress, a key contributor to the pathophysiology of β-thalassemia and SCD [81]. The findings of this analysis are partially supported by literature describing GWAS loci such as *FHIT*, *MMP26*, *ABCC1*, and *PFAS*, which have shown involvement in oxidative stress in various contexts. However, a detailed exploration of their potential roles in HbF regulation remains beyond the scope of this review.

The largest multi-ancestry GWAS for genetic modulators of HbF to date involving 28,279 participants was published by Cato et al. [48]. This study marks a significant advancement in the ability of GWAS to detect rare large-effect variations and identify novel candidate modifier genes for HbF, potentially paving the way for new therapeutic interventions in β hemoglobinopathies. However, with nearly 85% of participants from the general population (non-hemoglobinopathy) and a significantly smaller portion representing SCD, while β-thalassemia is nearly absent, there remains a considerable gap in research specific to β hemoglobinopathies. While preparing this manuscript and after completing the literature search, a U.S.-based GWAS on pediatric SCA patients (*n* = 467, 97% African American) was published, which identified a new genome-wide significant locus at 15q14 (rs8182015) in the *PGBD4*-*KATNBL1* intergenic region, and confirmed *BCL11A* (rs1427407 and rs766432) as a key modifier of HbF levels [82]. It is now crucial for global efforts to conduct large studies that represent diverse geographic locations and disease groups (SCD/thalassemia) to advance biomarker discovery. The primary aim of INHERENT [6] to genotype 30,000 hemoglobinopathy patients from diverse ethnic groups remains highly relevant as such large, multi-ethnic studies are essential for identifying and validating disease modifiers in the relevant patient populations. This can be used for patient stratification and personalized treatment.

To conclude, this systematic review has several strengths as it follows the recommendations for rigorous systematic review methodology. It employs a sensitive search strategy to identify all available relevant studies and extracts data in a standardized format, thus minimizing publication bias. Four independent reviewers completed the entire systematic review process, including title and abstract screening, full-text screening, data extraction, and quality assessment of the included studies. Furthermore, data was collated and presented in a clear tabular format and analyzed to describe existing knowledge while identifying areas that require improvement to pinpoint promising candidates for HbF re-activation as a therapeutic strategy for β hemoglobinopathies.

## Figures and Tables

**Table 1:** Keywords for each PICO element.

PICO Question Elements	Keywords
Population (P)	>2 years of age, males or females at birth, healthy (non-hemoglobinopathy) or with a β hemoglobinopathy, any ancestry group, any region across the world, with a measurable change in HbF levels or F-cell numbers.
Intervention (I)	GWAS-derived association of a sequence variation with HbF levels or F-cell numbers, i.e., the effect allele of a genetic polymorphism.
Comparison (C)	The other (non-effect) allele of a genetic polymorphism.
Outcome (O)	A measurable change in HbF levels or F-cell numbers.

**Table 2:** Characteristics of the included studies.

Study	Study Type	Population	Disease Status	Sample Size	Age Group	Trait
Menzel S et al., 2007 [17]	GWAS	European [TwinsUK]	non-Hbp	179	Child, Adult	F-cells
Uda M et al., 2008 [37]	GWAS	European [SardiNIA]	non-Hbp	4305	Child, Adult	HbF
Sedgewick AE et al., 2008 [18]	GWAS	African American [MSH] Chinese (Hong Kong)	SCA β-thalassemia trait	255 250	Adult Adult	HbF F-cells
Solovieff N et al., 2010 [39]	GWAS	African American [CSSCD; MSH; Duke University pHTN; BMC-pHTN] Thai Chinese (Hong Kong)	SCA β-thalassemia trait or HbE trait	848 (primary), 305 (replication) 113 406	Child, Adult Adult	HbF
Bhatnagar P et al., 2011 [38]	GWAS	African American [SITT]	SCD (SS, Sβ0)	440	Child	F-cells
Farrell JJ et al., 2011 [40]	GWAS	Chinese (Hong Kong)	β-thalassemia trait	659	Adult	HbF
Bae HT et al., 2012 [49]	Meta-analysis [GWAS for each cohort]	African American [CSSCD; PUSH; MSH; SITT; C-Data; Walk-PHaSST; Duke University OMG]	SCA	2040	Child, Adult	HbF
Milton JN et al., 2014 [41]	GWAS	African American [CSSCD; Walk-PHaSST; PUSH; C-Data]	SCD (SS, Sβ0)	841 (primary), 385 (replication)	Child, Adult	HbF
Mtatiro SN et al., 2014 [42]	GWAS	East African [MSC]	SCD (SS, Sβ0)	1213	Child	HbF
Danjou F et al., 2015 [43]	GWAS	European [SardiNIA]	non-Hbp	6602	Adult	HbF
Liu L et al., 2016 [35]	GWAS	African American [CSSCD; C-data; US-Thomas Jefferson University]	SCD (SS, Sβ0)	254	Child	HbF
Shaikho EM et al., 2017 [44]	GWAS	African American [CSSCD]	SCA	293	Child, Adult	HbF
Rampersaud E et al., 2021 [45]	GWAS	Unspecified [St. Jude SCCRIP/Baylor]	SCD (SS, Sβ0)	585	Child, Adult	HbF
Cato LD et al., 2023 [48]	Meta-analysis	European [Swedish; SardiNIA; UK-INTERVAL; GTEx; BIOS] Thai African [Tanzania; Walk-PHaSST; OMG-SCD; REDS-III Brazil; St. Jude SCCRIP/Baylor]	non-Hbp SCD	28,279	Child, Adult	HbF
Ojewunmi OO et al., 2023 [47]	GWAS	West African (Nigeria)	SCD (SS, Sβ0)	1006	Child, Adult	HbF
Hara Y et al., 2023 [46]	GWAS	European [UK-INTERVAL]	non-Hbp	11,004	Adult	HbF

**Population source/description**: BIOS, Biobank-based integrative omics study [50]; Chinese (Hong Kong) [51]; C-Data, Comprehensive Sickle Cell Centers Collaborative Data Project [52]; CSSCD, Cooperative Study of Sickle Cell Disease [53]; Duke University pHTN and BMC-pHTN [54,55]; GTEx, The Adult Genotype Tissue Expression Project [56]; MSC, Muhimbili Sickle Cohort [57]; MSH, Multicenter Study of Hydroxyurea in Patients With Sickle Cell Anemia [58,59]; REDS-III Brazil, Recipient Epidemiology Donor Evaluation Study (REDS)-III—Brazil Sickle Cell Disease Cohort [60]; SardiNIA [61]; SITT, Silent Cerebral Infarct Transfusion Multi-Center Clinical Trial [62]; St. Jude SCCRIP/Baylor [63]; OMG-SCD, Outcome Modifying Genes study [64]; PUSH, Pulmonary Hypertension, Hypoxia and Sickle Cell Disease [65]; TwinsUK [66]; UK-INTERVAL [67]; Walk-PHaSST, Sildenafil Therapy for Pulmonary Hypertension and Sickle Cell Disease [68]. **Abbreviations**: HbF, fetal hemoglobin; non-Hbp, non-hemoglobinopathy; SCA, sickle cell anemia; SCD, sickle cell disease.

**Table 3:** Risk of bias assessment using the Q-Genie tool.

	Rationale for Study	Selection and Definition of Outcome of Interest	Selection and Comparability of Comparison Groups	Technical Classification of the Exposure (Genetic Variant)	Non-Technical Classification of the Exposure (Genetic Variant)	Other Sources of Bias	Sample Size and Power	A Priori Planning of Analyses	Statistical Methods and Control of Confounding	Testing of Assumptions and Inferences for Genetic Analyses	Appropriateness of Inferences Drawn from Results
Menzel S et al., 2007 [17]					-	-					
Uda M et al., 2008 [37]			-		-	-					
Sedgewick AE et al., 2008 [18]					-	-					
Solovieff N et al., 2010 [39]			-		-	-					
Bhatnagar P et al., 2011 [38]			-		-	-					
Farrell JJ et al., 2011 [40]			-		-	-					
Bae HT et al., 2012 [49]			-	meta-analysis	-	-			meta-analysis	meta-analysis	
Milton JN et al., 2014 [41]			-		-	-					
Mtatiro SN et al., 2014 [42]			-		-	-					
Danjou F et al., 2015 [43]			-		-	-					
Liu L et al., 2016 [35]					-	-					
Shaikho EM et al., 2017 [44]					-	-					
Rampersaud E et al., 2021 [45]			-		-	-					
Cato LD et al., 2023 [48]				meta-analysis	-	-				meta-analysis	
Ojewunmi OO et al., 2023 [47]					-	-					
Hara Y et al., 2023 [46]					-	-					
**Color key**											
									-		
Some concerns	Low risk of bias				High risk of bias				N/A		

Domains of bias such as “non-technical classification of the exposure” and “other sources of bias” are marked as non-applicable (N/A) across all studies, as the GWAS methodology is specified and indicated for variant detection.

**Table 4:** List of QTLs mapped by GWAS-significant SNPs. The table illustrates genes and intergenic regions identified through GWAS-significant SNPs in a minimum of two independent studies. Apart from the Hara Y. et al. 2023 medRxiv study [46], PMIDs are provided. The table includes the number of reported gene-trait associations and the unique rs_IDs of associated SNPs. Abbreviations: Chr. (chromosome), non-Hbp (non-hemoglobinopathy), SCD (sickle cell disease), β-thal. trait (β-thalassemia trait).

Genes/Intergenic Sequence	Chr.	*n* Studies	PMID	*n* Gene-Trait Associations	*n* Unique rs_ID	Unique rs_ID	Disease
*BCL11A*	2	16	34283174, 27022141, 18245381, 38339995, 21326311, 22936743, 24585758, 20018918, 36993312, 28612458, 26366553, 17767159, 18691915, 21385855, 25372704	95	60	rs766432, rs10184550, rs6706648, rs6732518, rs6738440, rs10172646, rs4671393, rs7579014, rs7606173, rs10195871, rs11434093, rs11886868, rs1896295, rs1896296, rs58789059, rs66488669, rs72962585, rs72962586, rs72962592, rs72962596, rs72962602, rs72964414, rs72964419, rs7340264, rs75468792, rs7584113, rs766431, rs77434017, rs6709302, rs3771270, rs1123573, rs12477097, rs6545816, rs6545817, rs1427407, rs13019832, rs34211119, rs168565, rs45600937, rs45611631, rs7557939, rs114125602, rs11692396, rs45484694, rs45606437, rs4672393, rs555276704, rs79059225, rs1896294, rs17331129, rs7599488, rs12621957, rs971563, rs6729815, rs2665668, rs9789627, rs66808336, rs6739994, rs45527235, rs35125709	non-Hbp, SCD, β-thal. trait
*MIR4432HG*	2	3	36993312, 18245381, 17767159	15	14	rs1553934, rs112102583, rs243017, rs13027161, rs2137281, rs243027, rs243081, rs2540913, rs925483, rs243035, rs243038, rs181680, rs243073, rs243016	non-Hbp, SCD
*MIR4432HG-BCL11A*	2	3	21385855, 18245381, 36993312	16	16	rs1012585, rs12105503, rs12468946, rs12990774, rs2419919, rs10445937, rs11884411, rs12474693, rs888082, rs7599856, rs17027944, rs56207419, rs243058, rs243065, rs56317209, rs17028162	non-Hbp, SCD, β-thal. trait
*FANCL-MIR4432HG*	2	2	34283174, 36993312	5	5	rs2024410, rs2024411, rs2024412, rs79813524, rs243089	non-Hbp, SCD
*MIR4432HG;MIR4432*	2	2	36993312, 18245381	3	3	rs56168568, rs243078, rs243079	non-Hbp, SCD
*FHIT*	3	3	34283174, 24585758, 20018918	2	2	rs17062324, rs6446085	SCD
*CSNKA2IP-EPHA3*	3	2	34283174, 21326311	22	22	rs114652585, rs114769790, rs115269277, rs116495400, rs116738901, rs137965716, rs140300636, rs141149292, rs147271491, rs147692173, rs150254503, rs184058228, rs1845344, rs186261692, rs191926827, rs200688235, rs550429831, rs74504855, rs77318373, rs78355527, rs79855904, rs79950692	SCD
*CCNH-TMEM161B*	5	2	36993312, Hara Y.	4	3	rs57811114, rs1997323, rs62367635	non-Hbp, SCD
*HBS1L-MYB*	6	12	34283174, 38339995, 36993312, 21385855, 22936743, 26366553, 24585758, 25372704, 20018918, 17767159, 18245381, Hara Y.	63	41	rs34208856, rs35786788, rs61028892, rs66650371, rs9389268, rs9399137, rs9402686, rs1320963, rs1569534, rs4895441, rs6569992, rs6913541, rs7775698, rs9494139, rs1320959, rs1547247, rs2210366, rs56316290, rs6914717, rs6930223, rs9376090, rs114398597, rs114603312, rs115099895, rs116460276, rs148826327, rs1986846, rs9483788, rs9494142, rs9494145, rs7776054, rs9389272, rs13220662, rs9389263, rs55731938, rs7743042, rs9389266, rs76200612, rs6904897, rs9376092, rs9376095	non-Hbp, SCD, β-thal. trait
*ALDH8A1*	6	3	21385855, 36993312, Hara Y.	7	6	rs4646869, rs7772031, rs1022491, rs4646870, rs2142725, rs2072827	non-Hbp, SCD, β-thal. trait
*BACH2*	6	2	36993312, Hara Y.	5	4	rs2325259, rs4707609, rs1010474, rs3757247	non-Hbp, SCD
*HBS1L*	6	2	26366553, 36993312	3	3	rs11754265, rs61738647, rs41286240	non-Hbp, SCD
*RPS6KA2*	6	2	24585758, 20018918	1	1	rs6932510	SCD
*LINGO2-ACO1*	9	2	21326311, 25372704	2	2	rs16914695, rs62573842	SCD
*OR51B5*	11	6	36993312, 18245381, 34283174, 24585758, 20018918, 22936743	26	21	rs7482183, rs7113385, rs1391619, rs2340653, rs2647579, rs2647566, rs2736581, rs416586, rs7113817, rs10500635, rs1498473, rs2467218, rs2647593, rs369934, rs9665744, rs10838144, rs2736579, rs12291806, rs425610, rs10838102, rs4910785	non-Hbp, SCD
*HBB*	11	3	18245381, 36993312, 34283174	6	5	rs7936823, rs1003586, rs10742583, rs200771769, rs7946748	non-Hbp, SCD
*HBE1-OR51B4*	11	3	18245381, 36993312, Hara Y.	13	11	rs10768774, rs11036635, rs4910548, rs6578596, rs7119142, rs9633908, rs4910742, rs75356043, rs11036634, rs11036562, rs3888708	non-Hbp, SCD
*HBG1*	11	3	22936743, 24585758, 36993312	4	2	rs2855039, rs6578592	non-Hbp, SCD
*HBG2*	11	3	26366553, 18245381, 17767159	3	3	rs2855122, rs2855123, rs7482144	non-Hbp
*MMP26*	11	3	18245381, 34283174, 36993312	14	14	rs59973962, rs73405039, rs73405086, rs74052701, rs840716, rs4910694, rs11033556, rs114649801, rs12282202, rs840711, rs1368822, rs2596002, rs3850509, rs2595972	non-Hbp, SCD
*OR51B5;OR51B6*	11	3	22936743, 20018918, 24585758	13	7	rs4910755, rs4910756, rs5006884, rs5024042, rs7483122, rs5006883, rs4910752	SCD
*OR52A1-OR51V1*	11	2	36993312, 34283174	37	35	rs12788454, rs116780407, rs137944031, rs138266557, rs183055323, rs189023578, rs189088717, rs189324289, rs189408390, rs190486235, rs201061900, rs60603509, rs74234647, rs74683445, rs75037664, rs75351666, rs75696754, rs75753947, rs75852802, rs76164323, rs76219042, rs76535870, rs76561070, rs76821141, rs76955641, rs77221694, rs77632769, rs77742575, rs78948522, rs79026699, rs79194614, rs79261994, rs7479815, rs6578581, rs7105270	non-Hbp, SCD
*OR51B4-OR51B2*	11	2	34283174, 36993312	17	16	rs4910744, rs77311317, rs10605881, rs11036710, rs11036735, rs11036757, rs11036780, rs11036801, rs11826430, rs4910749, rs72886292, rs7482428, rs4459335, rs3886221, rs11036766, rs11036693	non-Hbp, SCD
*OR51B2-OR51B5*	11	2	34283174, 36993312	12	12	rs2723377, rs78428451, rs78930059, rs10160507, rs11036870, rs2723381, rs7481985, rs4269953, rs6578608, rs11036849, rs4271412, rs73404570	non-Hbp, SCD
*OR52E2-OR52A5*	11	2	34283174, 36993312	10	10	rs112284070, rs115241537, rs16909560, rs16909572, rs187058992, rs2499947, rs74656233, rs78801161, rs79983865, rs6578548	non-Hbp, SCD
*HBD-HBG1*	11	2	18245381, 36993312	9	7	rs3759073, rs3759074, rs4910736, rs2105819, rs4283007, rs968856, rs72869882	non-Hbp, SCD
*HBG2-HBE1*	11	2	18245381, 36993312	6	5	rs11036509, rs10160820, rs10768733, rs12284340, rs7480910	non-Hbp, SCD
*OR51V1-HBB*	11	2	18245381, 36993312	5	5	rs11036238, rs34220818, rs12805013, rs6421047, rs35207925	non-Hbp
*HBE1*	11	2	26366553, 36993312	4	4	rs7479652, rs67385638, rs72872548, rs3759067	non-Hbp, SCD
*MMP26;OR51G1*	11	2	18245381, 36993312	2	2	rs10836955, rs11034685	non-Hbp
*OR51B2*	11	2	34283174, 22936743	3	2	rs10837814, rs2723382	SCD
*OR51L1*	11	2	34283174, 36993312	2	2	rs16908208, rs2445292	non-Hbp, SCD
*HBBP1*	11	2	36993312, Hara Y.	3	1	rs10128556	non-Hbp
*COCH*	14	2	24585758, 20018918	1	1	rs2239580	SCD
*LRFN5-FSCB*	14	2	24585758, 20018918	1	1	rs17114175	SCD
*ABCC1*	16	2	36993312, Hara Y.	4	4	rs246232, rs60782127, rs165975, rs45521934	non-Hbp
*CTC1*	17	2	36993312, Hara Y.	6	6	rs529633547, rs4792621, rs8078338, rs3826543, rs8078338, rs8078338	non-Hbp, SCD
*PFAS*	17	2	36993312, Hara Y.	9	6	rs9902252, rs6503094, rs62637606, rs4791641, rs1132554, rs9891699	non-Hbp, SCD
*GCDH*	19	2	36993312, Hara Y.	3	3	rs1799918, rs11085825, rs11085824	non-Hbp, SCD
*NFIX*	19	2	26366553, 36993312	2	1	rs183437571	non-Hbp, SCD

## Data Availability

The original contributions presented in the study are included in the article/Supplementary Materials. Further inquiries can be directed to the corresponding author.

## Supplementary Materials

The supporting material for this article can be downloaded at: https://www.mdpi.com/article/10.3390/ijms252111408/s1.
