# Supplementary material for: Genetic Polymorphisms Associated with Fetal Hemoglobin (HbF) Levels and F-Cell Numbers: A Systematic Review of Genome-Wide Association Studies
Source: Int J Mol Sci. 2024 Oct 23;25(21):11408. doi: 10.3390/ijms252111408 (PMC11546522; doi:10.3390/ijms252111408)
Supplement: Supplementary file 1 [file ijms-25-11408-s001.zip › ijms-3221002-proof-supplementary figure.pdf]

International Journal of
Molecular Sciences

MDPI

Systematic Review

Genetic polymorphisms associated with fetal hemoglobin (HbF) levels and F-cell numbers: A systematic review of genome-wide association studies

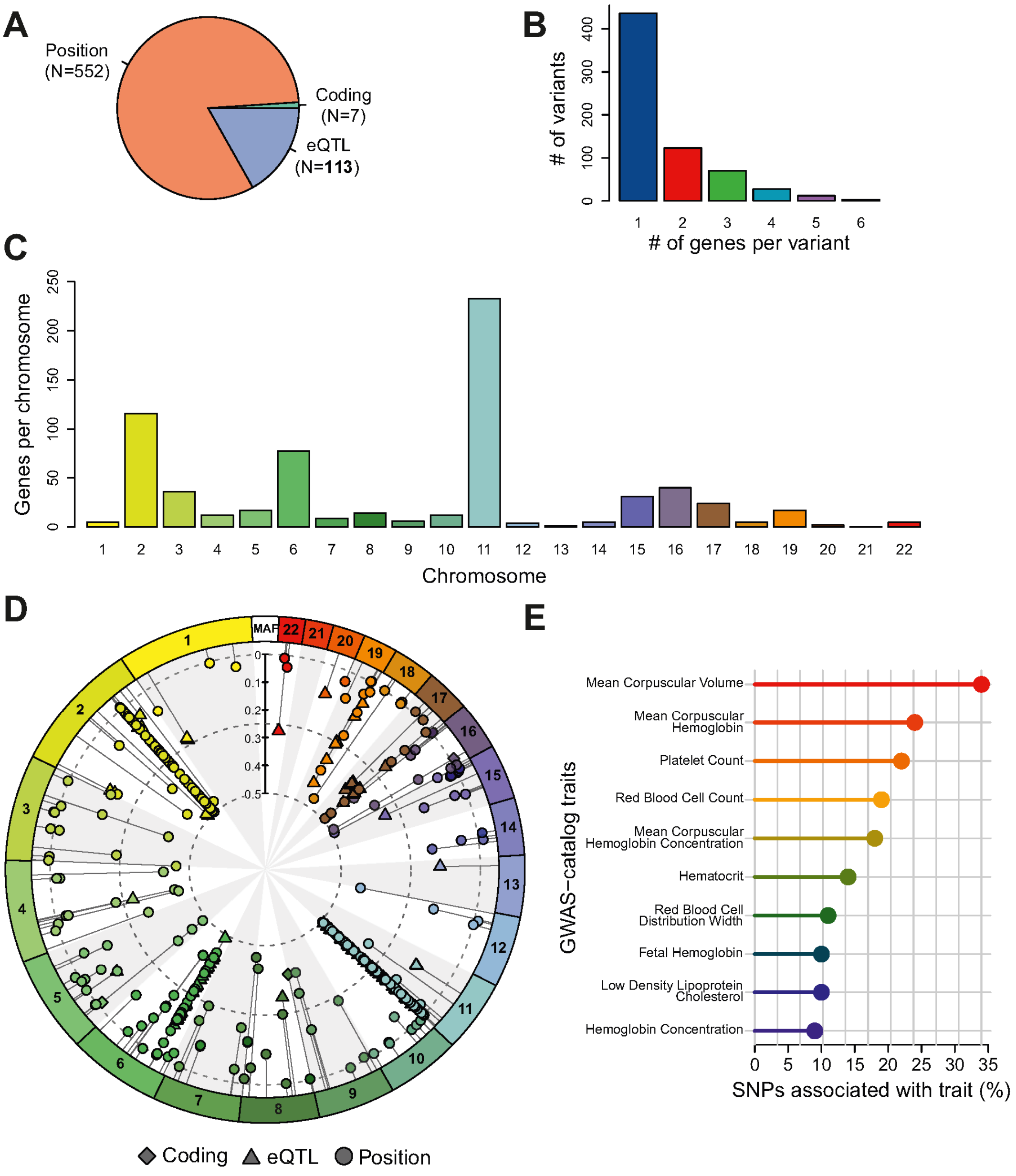

***Supplementary Materials Figure S1**. Gene mapping and functional annotation of the complete SNP set using snpXplorer. **A.** Pie plot shows variant annotations, classified as coding (green), eQTL (blue) or annotated by their genomic position (orange). **B.** Barplot shows the number of genes associated with each SNP. **C.** Plot shows the chromosomal distribution of SNPs. **D.** The circular summary figure shows the type of annotation of each SNP (as in plot 'A') as well as each SNP's minor allele frequency (MAF) and chromosomal distribution (as in plot 'C'). **E.** Fraction of SNPs associated with traits from the GWAS Catalog.*
